# Surgical resection for accessory spleen torsion: A case report

**DOI:** 10.1016/j.ijscr.2022.107835

**Published:** 2022-12-13

**Authors:** Masatsugu Kuroiwa, Hiroto Takayama, Yuji Uchikawa, Ryo Shimada

**Affiliations:** Azumino Red Cross Hospital, Nagano, Japan

**Keywords:** Accessory spleen, Torsion, Surgical treatment, Case report

## Abstract

**Introduction:**

Accessory spleen torsion is extremely rare, and surgery is often the emergency or elective treatment of choice.

**Presentation of case:**

A 20-year-old female with no specific medical history presented to our outpatient clinic with a chief complaint of abdominal pain. The patient was diagnosed with accessory spleen torsion by computed tomography. However, the abdominal symptoms and inflammatory reaction based on blood tests were mild, so a conservative treatment was selected. Subsequently, blood tests were normalized, and imaging studies showed that the accessory spleen was shrinking. Contrast-enhanced examination showed contrast enhancement in a portion of the infarcted accessory spleen region, indicating that the accessory spleen torsion had been released. Surgical resection was performed to prevent possible future re-torsion and hemorrhage of the accessory spleen.

**Discussion:**

The removed specimen seemed to be normal accessory spleen tissue with clear infarcted foci edges. This artery showed evidence of luminal organization and untwisting of the occluded artery.

**Conclusion:**

This accessory spleen torsion was treated conservatively; however, the patient was referred for surgical treatment.

## Highlights

• Surgery is often the treatment of choice for accessory spleen torsion.
• This is a rare case of an accessory spleen torsion.
• It highlights both conservative and surgical management.

## 1 Introduction

Accessory spleen is a congenital anomaly consisting of ectopic pancreatic tissue separated from the spleen [1]. Accessory spleen torsion becomes symptomatic after the vessels of the adnexa are twisted, impairing the blood flow to the spleen. Surgery is the treatment of choice in most cases [2]. This report presents a case of accessory spleen torsion for which conservative treatment was chosen; however, the patient underwent prophylactic surgical treatment once the torsion was resolved. This work has been reported in line with the SCARE criteria [3].

## 2 Presentation of case

**Fig. 1:**
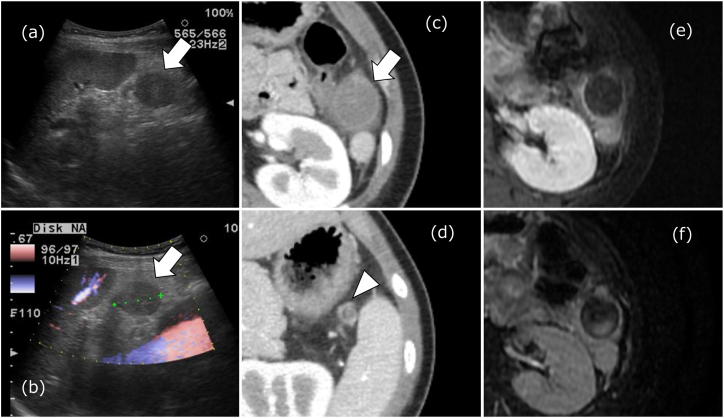
Imaging at the time of the first visit. (a) Ultrasonography showed an accessory spleen caudal to the spleen, appearing as a round mass approximately 26 mm in diameter (arrows). (b) The accessory spleen with no blood flow signal (arrows). (c) Contrast-enhanced computed tomography scan showed that the accessory spleen had slight dark staining at the margins, but other areas were poorly contrasted and showed evidence of accessory splenic infarction (arrows). (d) The accessory spleen showed a whirlpool sign with a continuous vascular structure from the splenic hilum (arrowhead). (e) Gadolinium magnetic resonance imaging showed no contrast effect in the accessory spleen. (f) Gadolinium magnetic resonance imaging showed low signal on T2-weighted imaging, indicating hemorrhagic necrosis.

**Fig. 2:**
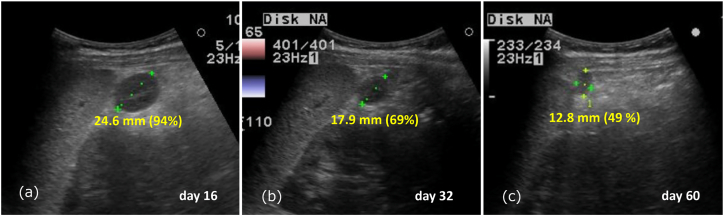
Ultrasound follow-up (a) The accessory spleen shrunk to 24.6 mm (94 %) and blood flow signals ceased at 16 days after the first visit. (b) The accessory spleen shrunk to 17.9 mm (96 %) at 32 days after the first visit. (c) The accessory spleen shrunk to 12.8 mm (49 %) at 60 days after the first visit.

**Fig. 3:**
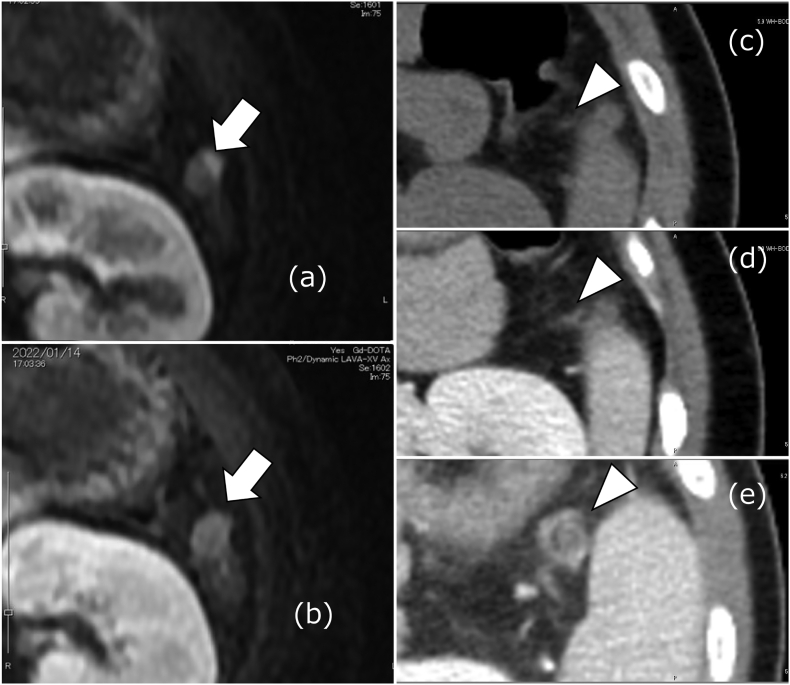
Computed tomography (CT) and magnetic resonance imaging (MRI) at 60 days from the first visit. (a, b) Gadolinium magnetic resonance imaging showed a contrast effect on a part of the accessory spleen and findings of restored blood flow to the accessory spleen (arrows). (c, d) Computed tomography scans showed that the whirlpool sign (arrowheads), observed two months earlier (e; arrowhead), had disappeared.

A 20-year-old female presented to the emergency department with upper abdominal and left back pain. The patient was diagnosed with acute gastroenteritis and treated conservatively with an intestinal regimen. Three days later, a computed tomography (CT) scan suggested accessory spleen torsion, and the patient was referred to our surgical department. Abdominal pain improved, and blood tests revealed hemoglobin (Hb) level was 124 g/L, CRP level was 11,300 μ0/L, and no major hematological abnormalities. An accessory spleen was visualized by ultrasonography (US) as a round mass, approximately 26 mm in diameter, caudally to the spleen, with no blood flow signal. There was also an increase in the fatty tissue hyperechoic region around the accessory spleen area, suggesting inflammation (Fig. 1a, b). Contrast-enhanced CT scans showed that the accessory spleen limbus had a slight dark stain, but other areas were poorly contrasted, suggesting infarction. The accessory spleen vascular structure was associated with the splenic hilum, showing a whirlpool sign (Fig. 1c, d). Gadolinium magnetic resonance imaging (Gd-MRI) showed a low signal on T2-weighted imaging (T2WI) with no contrast effect in the accessory spleen, indicating hemorrhagic necrosis (Fig. 1e, f). This patient was diagnosed as having accessory spleen torsion. Since the abdominal findings were mild and blood tests showed no major abnormalities, the patient was treated conservatively with acetaminophen 500 mg as an abortive measure. She was seen on days 16, 32, and 60 after discharge. Sixteen days after admission, the abdominal findings had resolved, blood tests normalized, and the accessory spleen had shrunk (Fig. 2). Two months later, Gd-MRI scans showed a contrast effect in a portion of the tortuous accessory spleen and findings of restored blood flow to the accessory spleen (Fig. 3a, b). The whirlpool sign had disappeared from the CT scans, and the torsion had resolved (Fig. 3c, d, e).

**Fig. 4:**
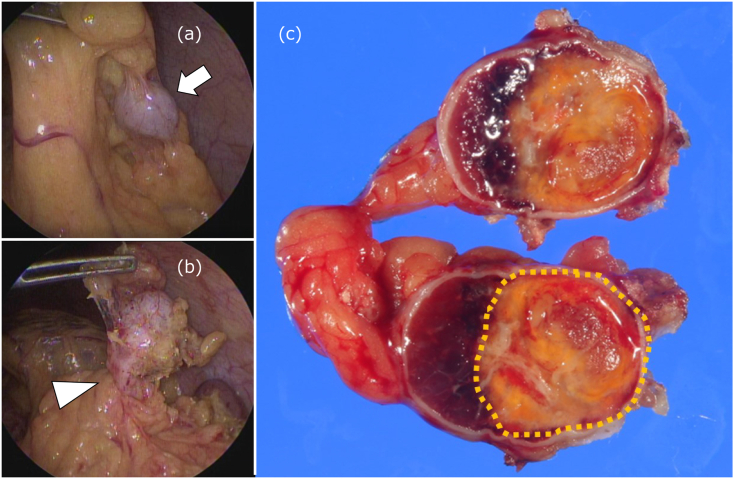
(a, b) Arrows: the accessory spleen; arrowhead: the vascular pedicle. (c) Dotted line: infarction with central necrotic tissue and granulation tissue formation.

Considering the risk of future re-torsion and bleeding, a prophylactic laparoscopic resection was performed in a right hemisagittal position, using a single port plus one. Intraoperative findings revealed the accessory spleen as a white nodule in the great omentum measuring approximately 20 mm in diameter. The accessory spleen was covered by the great omentum and adherent, suggesting a low risk of re-torsion. The adhesions were dissected, the vascular pattern was clipped, and the accessory spleen was removed (Fig. 4a, b). It was difficult to evaluate the tortuosity of the vascular pattern due to the surrounding adhesions. The operation time was 87 min. The patient was discharged on postoperative day four. Pathological examination revealed a 7 × 15 mm yellowish-toned nodule on the resection surface of the accessory spleen 24 mm from the edge; the accessory spleen revealed normal splenic tissue outside the nodule. The yellowish nodule had a central necrotic tissue area with granulation tissue formation, a finding suggestive of infarction (Fig. 4c). The artery on the infarcted focus side showed evidence of obstruction/occlusion and reopening.

## 3 Discussion

Accessory spleen is a congenital abnormality consisting of ectopic pancreatic tissue separate from the spleen [1], found in approximately 10–30 % of autopsied patients [4]. It is more common in females aged 20–40 and children younger than ten [5]. The accessory spleen can be observed on contrast-enhanced CT scans as a well-circumscribed, homogeneously contrast-enhanced round mass of under 2 cm.

The most common locations for the accessory spleen (22 %) are posteromedial to the spleen, anterolateral to the upper pole of the left kidney, and lateral, posterior, and superior to the pancreatic tail [6]. Approximately 43.3 % of the accessory spleen vascular pattern originates from the splenic artery [7]. Differential diagnoses of intra-abdominal masses with no clear vascular connection or tortuosity include mesenteric, intestinal stacked, and lymphatic cysts [8].

Accessory spleen torsion becomes symptomatic once the adnexa vessels are twisted, and blood flow to the accessory spleen is impaired. Unlike the gallbladder and gastrointestinal tract, the accessory spleen is a sterile organ with no adjacent organs, so it presents nonspecific symptoms (e.g., vague abdominal pain, nausea, vomiting, fever) when torsion occurs [2]. Accessory spleen torsion has been reported as a rare complication of intestinal obstruction and infection [9], [10]. In this case, the size of the accessory spleen at the time of resection was 24 mm. Termos et al. [11] found that accessory spleens larger than 6 cm were more prone to torsion. However, Ozeki et al. [12] reported torsion of a 30-mm accessory spleen. Susceptibility to torsion according to diameter remains an unresolved issue.

Accessory spleen infarctions appear during US examinations as subcapsular and triangular hypoechoic segmental lesions due to edema and necrosis, with the base parallel to the splenic periphery and the apex oriented outwards. The affected areas show no blood flow on color Doppler US [13]. Contrast-enhanced CT and MRI scans show necrosis following accessory spleen torsion as a slight peripheral or capsular contrast effect but no such effect inside the mass, with a twisted vascular pedicle connecting the accessory spleen to the splenic artery [12], [14]. MRI scintigraphy using ^99m^Tc-labeled phytic acid is said to be useful in functional accessory spleen diagnosis; however, it is unlikely to be used in an emergency because the radioactive agent is not taken up by the necrotic tissue caused by the torsion [15].

Asymptomatic accessory spleen does not require therapeutic intervention unless there is an underlying disease such as lymphoma, leukemia, thrombocytopenia, or hemosiderosis [16]; however, diagnosed accessory spleen torsion is generally treated by surgery. Cases operated on electively underwent surgery to diagnose tumors or an accessory spleen causing chronic abdominal pain, bowel obstruction or infection, or to manage the risk of bleeding or re-torsion. Scirè et al. [17] treated a 10-year-old male patient diagnosed with accessory spleen torsion with analgesics and antibiotics. The accessory spleen progressively reduced in dimensions when assessed at 3, 6, and 12 months. In our case, as in the report by Scire et al., symptoms resolved, blood tests normalized, and the accessory spleen shrunk. We opted for a conservative treatment because the patient was a young female. However, on the follow-up contrast examination, the torsion had resolved, the whirlpool sign disappeared, and the blood flow restored to part of the accessory spleen, suggesting a high risk of retorsion. Lhuaire [16] and Marsetti [18] report a case of accessory spleen with retorsion (Table. 1). The former was diagnosed with a renal cyst 24 years earlier and the latter with gallbladder edema 2 years earlier and was followed up. However, both were treated surgically due to persistent abdominal pain and were diagnosed with accessory spleen with retorsion. Therefore, in this case, we were concerned about the possibility of symptoms due to retorsion in the future, and surgical treatment was performed. The surgical findings indicated that the accessory spleen was fused to the omentum on its cephalic and caudal sides, making it unlikely to undergo torsion again. However, preoperative ultrasonography to evaluate adhesions could not determine the accessory spleen mobility. We speculated that the infarcted accessory spleen could become a peritoneal free body, potentially causing torsion and infarction of the peritoneal duct, aseptic fat necrosis, saponification and calcification of the fat content, and atrophy and shedding of the pedicle [19]. However, the removed specimen showed signs of arterial reopening or untwisting of the occluded artery on the infarcted focus side and risk of hemorrhage during future changes such as infarcted foci shedding. Conservative treatment for accessory spleen torsion may be indicated when (1) clinical findings and blood tests are mild; (2) there are no other complications; (3) there are no malignant findings. However, considering the risk of re-torsion, strict outpatient follow-up, including contrast studies, is necessary. Although more cases should be accumulated, surgical treatment should be considered once the torsion has been corrected and contrast recovery of the accessory spleen is observed.

**Table 1:** Previous reports of accessory spleen with retorsion.

Author	Year	Age	Sex	From onset to surgery (years)	Laboratory studies	Size(mm)	Preoperative diagnosed	Pathology
Lhuaire	2013	66	M	24	normal	30	Benign left renal cyst	Ischemic
Marsetti	1987	65	M	2	normal	110	Gallbladder edema	Necrosis

In terms of surgical technique, the free nature of the accessory spleen allows for easy dissection. Therefore, a single port or single port plus one is sufficient. We used a single port plus one in this case to prevent splenic injury due to intraoperative manipulation. The patient was positioned in the right hemisagittal position to secure an adequate visual field.

## 4 Conclusion

We reported a rare case of accessory spleen torsion. The treatment plan should be based on abdominal findings, blood tests, and complications. Contrast studies should be performed if electing for conservative treatment. A single port or a single port plus one is sufficient if surgery is performed.

## Ethics approval and consent to participate

Ethics approval/consent was waived.

## Consent to publication

Written informed consent was obtained from the patient for publication of this case report and accompanying images. A copy of the written consent is available for review by the Editor-in-Chief of this journal on request.

## Funding

This research did not receive any specific grant from funding agencies in the public, commercial, or not-for-profit sectors.

## CRediT authorship contribution statement

MK acquired and interpreted the data and drafted the article. HT edited the article and participated in the study design and revision of the manuscript. All authors read and approved the final manuscript.

## Authors' information

Not applicable.

## Registration of research studies

1. Name of the registry: N/A
2. Unique identifying number or registration ID: N/A
3. Hyperlink to your specific registration (must be publicly accessible and will be checked): N/A

## Guarantor

Masatsugu Kuroiwa

## Declaration of competing interest

There are no conflicts of interest to declare.

## Data Availability

Data sharing is not applicable to this article as no datasets were generated or analyzed during the current study.
